# Sensory epithelia of the fish inner ear in 3D: studied with high-resolution contrast enhanced microCT

**DOI:** 10.1186/1742-9994-10-63

**Published:** 2013-10-27

**Authors:** Tanja Schulz-Mirbach, Martin Heß, Brian D Metscher

**Affiliations:** 1 Department of Biology II, Zoology, Ludwig-Maximilians-University, Martinsried, Germany; 2 Department of Theoretical Biology, University of Vienna, Vienna, Austria

**Keywords:** microCT, Interactive 3D models, Fish inner ear, Macula, Sensory epithelium

## Abstract

**Introduction:**

While a number of studies have illustrated and analyzed 3D models of inner ears in higher vertebrates, inner ears in fishes have rarely been investigated in 3D, especially with regard to the sensory epithelia of the end organs, the maculae. It has been suggested that the 3D curvature of these maculae may also play an important role in hearing abilities in fishes. We therefore set out to develop a fast and reliable approach for detailed 3D visualization of whole inner ears as well as maculae.

**Results:**

High-resolution microCT imaging of black mollies *Poecilia* sp. (Poeciliidae, Teleostei) and *Steatocranus tinanti* (Cichlidae, Teleostei) stained with phosphotungstic acid (PTA) resulted in good tissue contrast, enabling us to perform a reliable 3D reconstruction of all three sensory maculae of the inner ears. Comparison with maculae that have been 3D reconstructed based on histological serial sections and phalloidin-stained maculae showed high congruence in overall shape of the maculae studied here.

**Conclusions:**

PTA staining and subsequent high-resolution contrast enhanced microCT imaging is a powerful method to obtain 3D models of fish inner ears and maculae in a fast and more reliable manner. Future studies investigating functional morphology, phylogenetic potential of inner ear features, or evolution of hearing and inner ear specialization in fishes may benefit from the use of 3D models of inner ears and maculae.

## Introduction

Inner ears in modern bony fishes (Teleostei) are composed of three semicircular canals and three otolithic end organs. Sensory epithelia of the canals, called cristae, are solely overlain by a gelatinous matrix, the cupula. In each of the end organs, the sensory epithelium (macula) is overlain by a massive calcium carbonate biomineralisate, the otolith. Maculae are characterized by orientation patterns of ciliary bundles of the sensory hair cells, i.e. the morphologically and physiologically polarized ciliary bundles form differently oriented groups on the macula. The orientation of a ciliary bundle is determined by the position of its eccentrically placed kinocilium relative to the stereocilia.

Teleosts show several different orientation patterns, and these patterns have been interpreted with regard to or correlated with acoustic abilities (e.g. sound source localization) of fishes (e.g., [1]). For evaluation of patterns, maculae are flattened during preparation for further analysis using scanning electron or confocal microscopes (e.g., [2]). This in turn means that orientation patterns are obtained, shown, and compared between species in a purely two dimensionally way irrespective of the natural curvature of the maculae in the ear. As has already been stated by Platt and Popper (1981) [3], orientation patterns should preferably take into account the three dimensional structure of sensory epithelia. Until now, the reliable reconstruction of maculae in their original 3D curvature was difficult and has to our knowledge only rarely been performed (zebrafish: [4]; Atlantic molly, *Poecilia mexicana*: [2]). These reconstructions based on histological serial sections were labor-intensive and results were in part uneven as maculae were prone to distortion or artifacts such as disruption during dehydration or embedding procedures.

The non-destructive imaging method of x-ray micro-computed tomography (microCT) was used in a recent study on cichlid ears [5] and in an investigation of 3D utricular and saccular macula in mammals [6]. These studies indicate that a rather fast 3D reconstruction should be possible using microCT. However, iodine stained samples of cichlids [5] did not provide a sufficient contrast to discriminate otolith, otolithic membrane, maculae, and innervating nerve fibers of the VIII^th^ cranial nerve. The aim of our study therefore was to establish a fast and more reliable method to three-dimensionally reconstruct whole inner ears of fishes with special focus on the reconstruction of the 3D curvature of maculae.

Our results indicate that reliable 3D reconstructions of inner ears including otoliths and maculae can be obtained by using high-resolution microCT imaging of specimens stained with phosphotungstic acid.

## Methods

### Study animals

We chose two species from two different orders within the teleosts, the cyprinodontiform black molly *Poecilia* sp. (Poeciliidae, live-bearers; four specimens) and the perciform slender lion head cichlid *Steatocranus tinanti* (Cichlidae; two specimens) from local aquarium stores because inner ear morphology including otoliths and/or sensory epithelia of *S. tinanti* and of the closely related species *Poecilia mexicana*, respectively, have been investigated in previous studies [2,5] and could thus be used for comparative purposes.

### PTA staining

Specimens were anesthetized and euthanized with an overdose (approx. 0.02%) of MS 222 (ethyl 3-aminobenzoate methanesulfonate; Sigma-Aldrich). Subsequently, scales on the head and in the region of the shoulder girdle were removed and the abdomen was ventrally opened to facilitate penetration of the fixative and the staining solution. Fishes were then fixed in 10% buffered formalin (buffered in 0.1 M cacodylate buffer, pH = 7.2) for up to five days at 4°C. Cacodylate buffer was used instead of phosphate buffer as with the latter phosphate crystals often grow during fixation nearby or on the surface of otoliths (pers. observ. TSM). Based on our previous experience of weak staining of inner ears for microCT with Lugol’s solution (aqueous iodine/iodide staining) and on comparative studies on different staining agents for microCT investigations [7,8], we chose phosphotungstic acid (PTA; Sigma-Aldrich P4006) because it has been observed to stain other sensory epithelia well such as olfactory epithelia in fishes ([7]; unpublished observations). Prior to staining, a microCT scan was performed using the unstained samples still in aqueous fixative solution.

**Figure 1:**
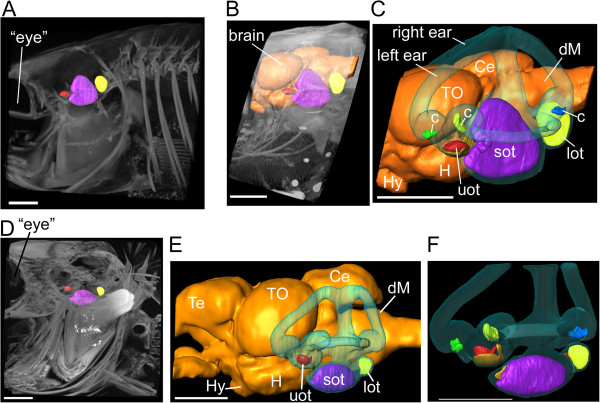
**Comparison of volume renderings based on tomographic reconstructions of the posterior part of the fish head of a black molly (A-C) and *S. tinanti *(D-F), including the ears of the same specimens before (A, D) and after phosphotungstic acid staining (B-C, E-F).** Without contrast enhancement, only bones and otoliths can be visualized **(A, D)**. Phosphotungstic acid distinctly enhances tissue x-ray contrast enabling 3D reconstructions of the inner ears including the sensory epithelia (maculae and cristae) **(C, F)**. Brain (orange): Ce, cerebellum; dM, dorsal medulla; Hy, hypophysis; H, hypothalamus; TO, tectum opticum; ears (transparent blue): c, cristae (green, yellow, blue); lot, lagenar otolith (yellow); sot, saccular otolith (purple); uot, utricular otolith (red). Scale bars, 1 mm.

One black molly specimen (Figure 1A-C) was dehydrated through an ascending ethanol series (30%, 50%, 70%) and then transferred to 0.3% PTA in 70% ethanol for six days. After staining it was returned to 70% ethanol for scanning and storage. The other samples were dehydrated to methanol (50%, 75%, 100%, at least 1 hour each) and were stained in 1% PTA in 100% methanol for four days. Samples were transferred back to methanol for scanning and storage.

To check the effects of dehydration and staining, one individual of each species was scanned as (1) non-dehydrated and unstained, (2) dehydrated and unstained, and (3) dehydrated and stained sample to test whether otoliths were partly dissolved or affected (e.g. growth of phosphate crystals) by the staining using PTA in methanol. A first scan was made of each specimen while in the fixative solution. Subsequently, specimens were dehydrated through an ascending methanol series (50%, 75%, 100%; at least one hour per step and overnight in 100%). Prior to staining, a second scan was performed using the unstained and dehydrated sample to check for potential effects of dehydration, i.e. shrinkage and/or distortion. Fishes were then transferred to 1% PTA in 100% methanol for four days. The staining solution was then changed to 100% methanol, in which the sample was scanned.

Testing the dehydration and staining on two spare black molly specimens (33 and 35 mm SL, 5 and 6 mm lateral body thickness) showed that overnight staining (approx. 18 hours) in 1% PTA in methanol was insufficient for full penetration and contrasting of the internal structures, and 48 hours of staining provided some contrast of the peripheral body, but not the central portions (data not shown). Four days of staining proved consistently adequate for fish specimens of this size, and this was adopted a general minimum staining time.

### MicroCT

Scans of inner ears with special focus on sensory epithelia were performed with a MicroXCT high-resolution microCT system (Xradia, MicroXCT-200, Zeiss X-Ray Microscopy, Pleasanton, CA) with a tungsten microfocus X-ray source and variable secondary optical magnification. These scans were made with an anode voltage setting of 40 kV at 4-8 W, with an exposure time of 5–8 seconds and 2x2 pixel binning for projection images every 0.25°. Reconstructed image stacks had isotropic voxel sizes of 9.4 μm (unstained), 5.4 μm (stained), or 2.0 μm (stained, high-resolution scan) for the black molly (Figure 1A-C) and 10 μm (stained) and 4 μm (stained, high-resolution scan) for *S. tinanti* (Figure 1D-F).

Scans checking potential artifacts of dehydration or staining were always performed using the same isotropic voxel size of 10 μm for both specimens (one specimen per species) and all three conditions (non-dehydrated and unstained; dehydrated and unstained; dehydrated and stained).

### 3D reconstructions

3D renderings of inner ears including otoliths, sensory epithelia such as maculae and cristae, and the posterior part of the brain were performed in AMIRA® v. 5.4.1 (Visage Imaging GmbH, Berlin, Germany).

Mainly a threshold-based segmentation was applied and, if necessary this labeling was refined or corrected using the brush tool. This means that after setting a threshold which corresponds a certain grey value, only voxels with values greater than or equal this threshold were assigned to the respective LabelField of the structure of interest (otolith, brain, etc.). For the reconstruction of otoliths and organs, initially every 5th to 10th image was labeled, with subsequent interpolation of structures on intervening images. Using the interpolate function may save time and is important to obtain rather smooth surfaces right away [9]. Ruthensteiner (2008) [9] recommended initial labeling of every third image; as otoliths and most of the organs labeled in our samples changed gradually across slices, we found labeling of every 5th (otoliths, labyrinth, sensory epithelia) to 10th (brain) image sufficient to yield a first good approximation of the respective structure. Subsequently, we checked every image for potential interpolation errors such as still unlabeled portions of the respective structure or labeled regions belonging to another organ. These errors were corrected using the brush tool by labeling or removing erroneously labeled parts where necessary.

Subsequently, every otolith and organ was separated from the 'master’ LabelField file into single LabelFields and saved as separate files. In the case of checking potential artifacts of dehydration or staining, the 'master’ LabelField was directly used for surface rendering without prior separation of single LabelFields. Surface rendering was performed with the SurfaceGen module. If necessary surfaces of each labeled object were reduced to 100,000 surfaces. This was followed by the smoothing of surfaces using the SmoothSurface module (20 iterations; unconstrained smoothing).

### Interactive 3D models

In order to give a full 3D impression of reconstructed structures, we created interactive figures in which all organs can be interactively accessed (free choice of perspective and organ composition) and thus provide a better understanding of the spatial relationship of maculae to each other as well as the amount of their curvature. Interactive 3D pdf models were created using Adobe Acrobat 9 pro extended (Adobe Systems, San Jose, CA, USA) and Deep Exploration 5.5 (right hemisphere) modifying the procedure described by Ruthensteiner and Heß (2008) [10].

### Validation of 3D reconstructions

The right inner ear of one black molly and the left inner ear of one *S. tinanti* were dissected out, semicircular canals were removed, and end organs were captured in (antero-)medial or ventral (utricle of the *S. tinanti* specimen) views while taking a stack of 8–13 images at different focal planes for each view using a Leica MZ 16 F stereomicroscope equipped with a ProgRes® C5 camera, applying the ProgRes® MAC CapturePro 2.7.6 image capture software (Jenoptik AG, Germany). To combine the in-focus areas of the source images to an extended-focus 2D-image, we created a montage image using the “Do stack” tool in CombineZM (Image Stacking Software by Alan Hadley, UK).

Moreover, we compared the 3D reconstructed macula sacculi and macula lagenae of the specimen studied here with the respective maculae of *Poecilia mexicana* described in detail in a previous study by TSM and MH [2,5].

### Evaluation of ciliary bundle orientation in 2D

To validate 3D reconstructed maculae of *S. tinanti* and to illustrate one possible application of 3D models of sensory epithelia, we first determined orientation patterns of ciliary bundles of the maculae of *S. tinanti* in 2D. In the case of the black molly, orientation patterns of ciliary bundles of the closely related *P. mexicana* were available from a previous study [2]. In both species, the macula utriculi showed the strongest three-dimensional curvature. We therefore chose this macula type to project the 2D orientation patterns of ciliary bundles onto the respective 3D models.

Immunohistochemistry: To identify orientation patterns, ciliary bundles of the maculae in eight specimens of *S. tinanti* (SL: 43–83 mm) were stained according to the method introduced by Lu and Popper (1998) [11] with TRITC-labeled phalloidin (Sigma-Aldrich, St. Louis, MO, U.S.A.) for stereocilia and anti-bovine α-tubulin mouse monoclonal antibodies (Molecular Probes®, Invitrogen, Darmstadt, Germany) and Alexa Fluor 488 conjugated anti-mouse secondary antibodies (Molecular Probes®, Eugene, OR, U.S.A.) for kinocilia. Prior to staining, the fresh heads were fixed for 1–2 hours in 10% buffered (0.1 M phosphate buffer) formalin solution at room temperature. Inner ears were dissected out in fixative, otoliths removed, and then sensory epithelia were washed four times in 0.1 M phosphate buffer with 0.01% sodium azide at 20 minute intervals at room temperature on a slowly moving shaker. All further steps (unless specified otherwise) were performed at room temperature on a shaker, and after every staining/antibody step tissue samples were washed four times with phosphate buffer with sodium azide at 20 minute intervals. Inner ears were incubated in blocking solution for 1 h and then incubated overnight in anti-bovine α-tubulin mouse monoclonal antibodies (1:200 dilution in 0.1 M phosphate buffer with sodium azide). Sensory epithelia were incubated in Alexa Fluor 488 anti-mouse antibodies (1:200 dilution in 0.1 M phosphate buffer) for 1.5 h at 37°C and in TRITC-labeled phalloidin (1:100 dilution in 0.1 M phosphate buffer) for 4 to 5 h. After the staining procedure, samples were stored at 4°C for one day, after which the sensory epithelia were mounted on a slide with an anti-fading medium, VectaShield® (Vector Laboratories). In this medium, the sensory epithelia were carefully flattened and then covered with a cover slip, sealed with nail polish, and stored at 4°C.

Confocal imaging: Samples were investigated with a Leica TCS SP2 inverse confocal laser scanning microscope (CLSM) using a Leica HCX PL APO UV 40x oil dipping objective (NA = 1.25) and with the 488 nm argon-gas-laser line and the 543 nm He-Ne laser. Overlapping image stacks of each macula were photographed at a z-step size of 1 μm and a pixel size of 183 nm x 183 nm. The image stacks were reduced to one image by applying the brightest point projection tool in Image J v. 1.46r. Brightest point projected images were used for creating one map of the stained stereocilia of each macula in Adobe Photoshop CS4®. Orientations of ciliary bundles were determined as described by Lu and Popper (1998) [11].

### Projection of 2D orientation patterns on the 3D models of the maculae

In AMIRA® v. 5.4.1 created and rendered surfaces were opened in Adobe Photoshop CS4® Extended as wavefront (obj) files. Then arrows indicating the orientation of groups of ciliary bundles were drawn on the dorsal face of the 3D models of the macula utriculi of *S. tinanti* and *Poecilia* sp., respectively.

## Results and discussion

### Effects of dehydration and staining

**Figure 2:**
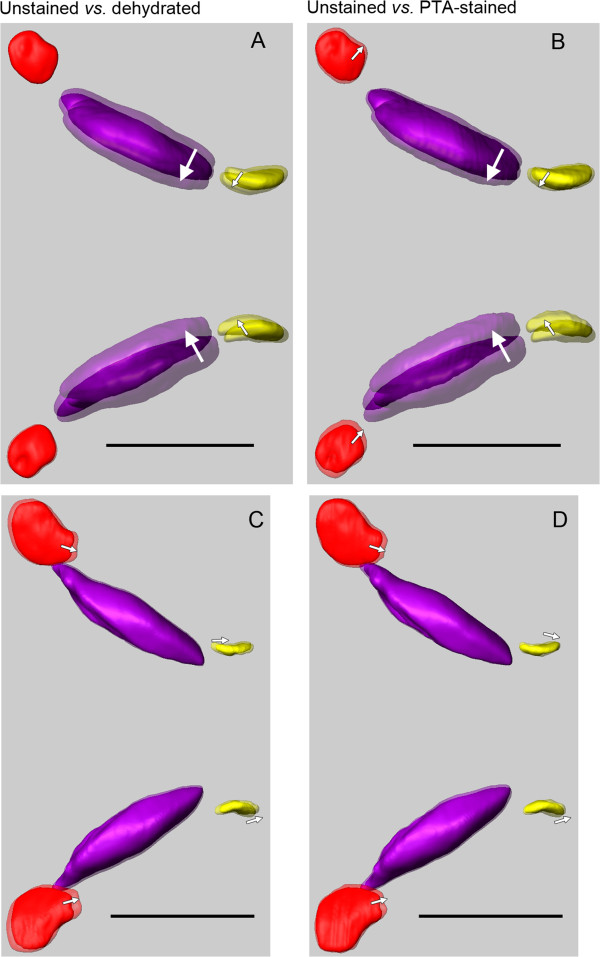
**Effects of dehydration and staining on inner ears illustrated by the position of the otoliths.** In dorsal view, 3D reconstructed utricular (red), saccular (purple), and lagenar otoliths (yellow) show a discernible shift in the black molly **(A-B)** provoked by dehydration and staining while in *S. tinanti***(C-D)** the treatment effects are small. Scale bars, 1 mm.

**Figure 3:**
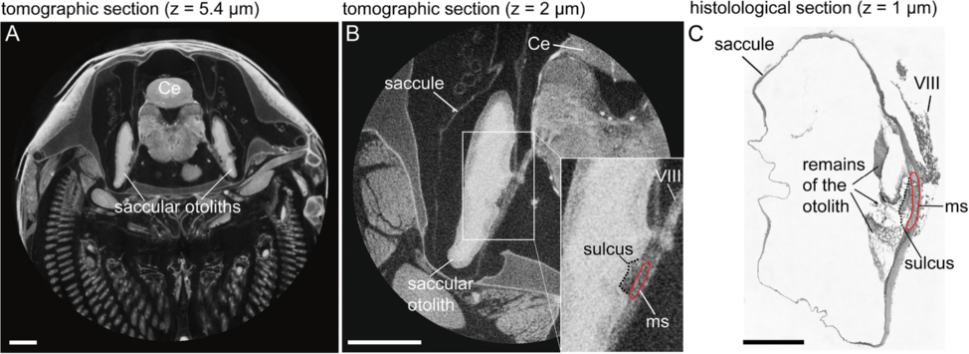
**Transverse tomographic (A-B) and histological sections (C) displaying the *Poecilia *saccule, its otolith and the corresponding macula.** The histological section **(C)** is from the inner ear of *Poecilia mexicana*. The higher resolution tomographic section in **(B)** shows that the nerve (VIII), otolith, and macula of the saccule could effectively be discriminated. For identification of the delimitations of the macula, the clearly visible sulcus of the otolith in the tomographic sections could be used; the sulcus is the furrow on the medial face of the saccular otolith housing the macula sacculi. Despite better discrimination of the macula from overlying otolithic membrane and the underlying basal lamina in the histological section, delimitations are still difficult to identify because of the gradual transition of the macula into non-sensory epithelium and the few organic remains of the dissolved otolith. In the insert in **(B)** and in **(C)** the sulcus is indicated by a dotted line and the macula is red labeled. Ce, cerebellum; ms, macula sacculi; VIII, part of the VIII^th^ cranial nerve innervating the macula sacculi. Scale bars, 500 μm.

The overlays in Figure 2 show that dehydration and staining led to a discernible shift of otoliths in the black molly mainly towards the brain (Figure 2A-B). Dehydration resulted in an inward tilt of saccular and lagenar otoliths whereas the utricular otoliths were still in place (Figure 2A). Staining seemed to affect the utricular otoliths that both shifted slightly into right lateral position (Figure 2B). In *S. tinanti* effects of dehydration and staining were weak resulting in a faint posterior shift of utricular and lagenar otoliths (Figure 2C-D). Thus, PTA staining seems to affect inner ears to different amounts depending on the fish species under study. The stronger effect in the black molly may be explained by the fact that the bony lamella partly surrounding the medial portions of the saccule and lagena in *S. tinanti* is almost completely absent (see Figure 3A). Such effects must be taken into account when interpreting the spatial relationship of the maculae to each other; while it is unlikely that the curvature of the maculae themselves is affected because the overlying otoliths which stabilize the maculae displayed the same morphology irrespective from the treatment applied (Figures 1A *vs.* B-C and D *vs.* E-F; 2A, C *vs.* B, D; 4A-B *vs.* C). Moreover, all reconstructed maculae of the black molly and *S. tinanti* showed a continuous undistorted curvature (Additional file 1, Additional file 2).

**Figure 4:**
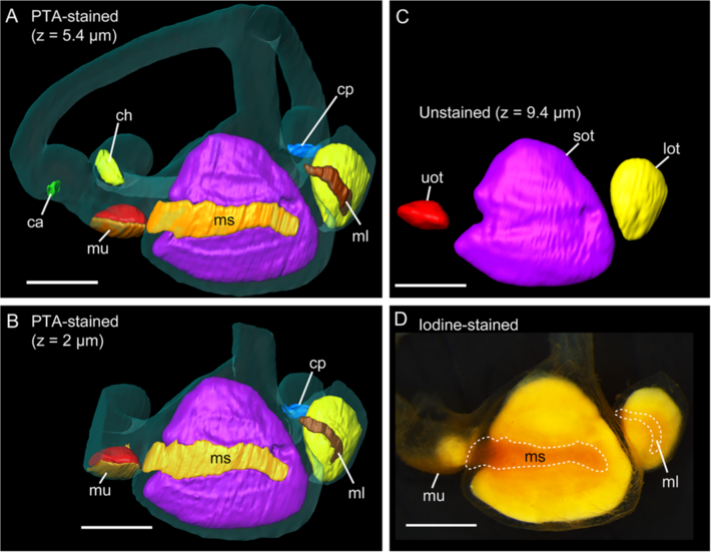
**High-resolution contrast enhanced microCT imaging allows accurate reconstruction of the maculae in the black molly.** Accuracy of reconstruction of the macula lagenae was especially improved by using the image stack scanned with higher resolution **(B***vs.***A)**. Comparison with the dissected right ear **(D)** shows that end organs, maculae (especially the macula sacculi), and otoliths were reliably reconstructed. Note that in **(B)** the semicircular canals and the utricle are incompletely reconstructed because the field of view during scanning had to be restricted to achieve the higher resolution. For a better comparison with **(B)**, semicircular canals including the ampullae were removed from the iodine stained, dissected ear **(D)**. **(C)** shows the reconstructed otoliths of the specimen prior to staining. c, cristae of the anterior (ca, green), horizontal (ch, yellow), and posterior (cp, blue) semicircular canals; lot, lagenar otolith (yellow); ml, macula lagenae (dark brown); ms, macula sacculi (yellow orange); mu, macula utriculi (light brown); sot, saccular otolith (purple); uot, utricular otolith (red). Iodine stained otoliths all have a yellowish appearance while maculae and the overlying as well as surrounding otolithic membranes are orange red. Scale bars, 500 μm.

**Figure 5:**
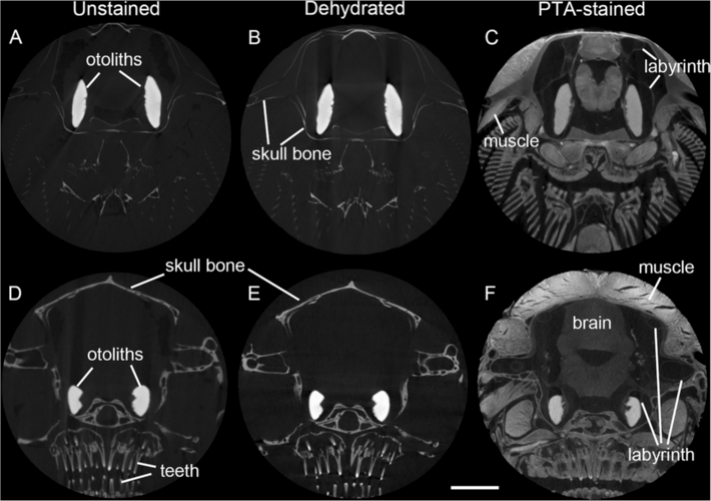
**Enhancement of tissue contrast by performing PTA staining.** In transverse tomographic sections of the unstained **(A, D)** or unstained and dehydrated samples **(B, E)** of both species **(A-C**, black molly; **D-F**, *S. tinanti***)** only bone, pharyngeal teeth and otoliths can easily be identified while after staining **(C, F)** diverse other tissues and organs such as brain, muscles, and the membranous labyrinth are now clearly visible. All scans were performed using the same isotropic voxel size of 10 μm for both specimens and all three conditions. Scale bar, 1 mm.

A comparison of unstained, unstained and dehydrated *vs.* PTA stained tomographic sections displays a clear enhancement of the tissue contrast in both species when performing the staining. While in the unstained sections only bone, pharyngeal teeth and otoliths are clearly visible (Figure 5A-B, D-E), the sections of the PTA-stained samples allow the identification of diverse tissues, e.g. brain, muscles, and the membranous labyrinth (shown by the saccule and parts of the semicircular canals) (Figure 5C, F).

### Reliability of 3D reconstructed maculae

**Figure 6:**
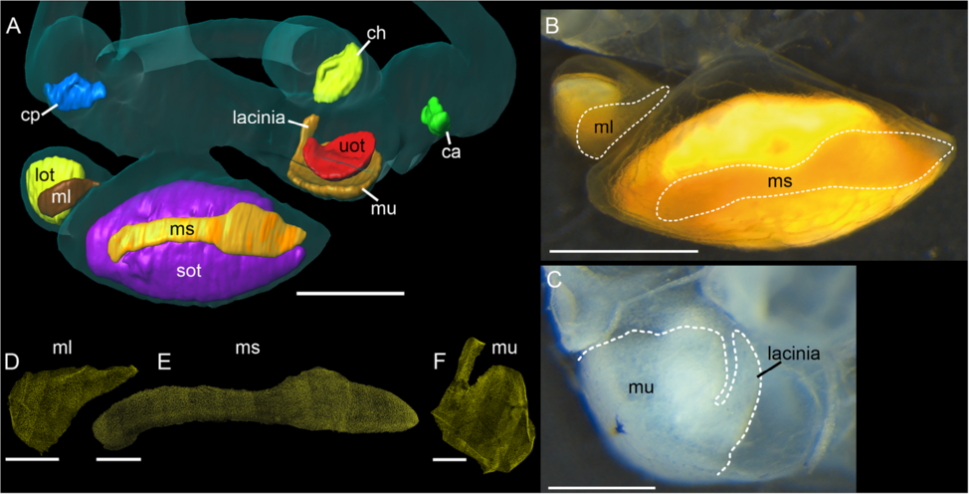
**High-resolution contrast enhanced microCT imaging allows accurate reconstruction of the maculae in *S. tinanti*.** Comparison with the dissected left saccule, lagena **(B**; anteromedial view**)**, and the utricle **(C**; ventral view**)** and the phalloidin-stained maculae **(D-F)** shows that end organs, maculae (especially the macula utriculi), and otoliths were reliably reconstructed **(A)**. For a better comparison, semicircular canals including the ampullae were removed from the iodine stained, dissected saccule and lagena **(B)**. ca, anterior semicircular canal; ch, horizontal semicircular canal; cp, posterior semicircular canal; lot, lagenar otolith; ml, macula lagenae; ms, macula sacculi; mu, macula utriculi; sot, saccular otolith; uot, utricular otolith. **(A-B)**: Scale bars, 500 μm; **(C-F)**: Scale bars, 250 μm.

**Figure 7:**
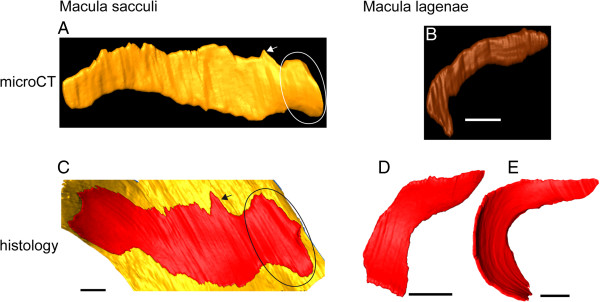
**Validation of the 3D reconstructed maculae based on microCT imaging**. 3D reconstructed macula sacculi **(A)** and macula lagenae **(B)** of the herein studied black molly are compared with a corresponding reconstruction from the Atlantic molly *Poecilia mexicana* in a previous study [2]. Reconstructions of the maculae of P. mexicana **(C-E)** based on histological serial sections (thickness of sections: 1 μm). The shape of the respective macula types of black molly and Atlantic molly show a high degree of similarity, especially with regard to the macula sacculi, indicating the reliability of 3D reconstructed sensory epithelia based on microCT imaging. Similar features of the maculae sacculi are marked by the arrow and circle. The yellow area in **(C)** represents reconstructed non-sensory epithelium surrounding the macula sacculi. All maculae are shown in lateral view with their dorsal side up and anterior to the right. Scale bars, 100 μm.

PTA staining allowed for a good 3D reconstruction of sensory epithelia, especially regarding the macula sacculi and the macula utriculi, when comparing with the maculae of the dissected inner ears (black molly: Figure 4A-B *vs.* D; *S. tinanti*: Figure 6A *vs.* B-C). In *S. tinanti*, the 3D reconstructed maculae showed high congruence with those stained with TRITC-labeled phalloidin (Figure 6A *vs.* D-F). The result was refined and improved in the black molly using higher resolution images for the reconstruction of the macula lagenae (Figure 4A *vs.* B) and of the lateral portion of the macula utriculi, namely the lacinia (Additional file 1, inner ear in lateral view). A comparison between the reconstructed macula sacculi of the black molly (*Poecilia* sp.) and that of *P. mexicana* (studied by Schulz-Mirbach et al. 2011 [2]) showed several similarities such as the dorsal bulge in the anterior portion of the macula and the overall shape of the anteriormost part (Figure 7A, C) that is not overlain by the saccular otolith. The macula lagenae was also similar in its overall shape and curvature to the maculae lagenae from *P. mexicana*; the ventral portion, however, was distinctly narrower in the herein reconstructed macula (Figure 7B, D-E).

**Figure 8:**
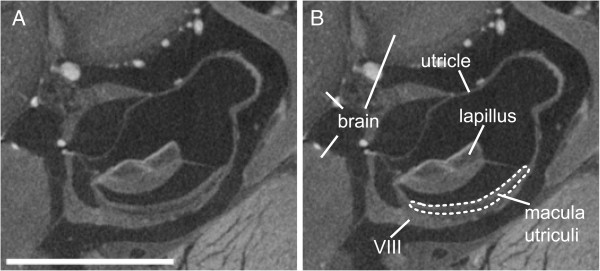
**Transverse tomographic section displaying the utricle, its otolith, and the corresponding macula of *S. tinanti*.** In **(A)** the section is shown without labeling and in **(B)** the same section is illustrated indicating the delimitations of the macula utriculi. The macula utriculi can clearly be distinguished from the part of the VIII^th^ nerve innervating the macula. Scale bar, 500 μm.

In order to derive reliable results for the 3D reconstruction of the maculae, it is necessary to incorporate prior knowledge of the shape of sensory epithelia and the inner ear anatomy of the species under study based on dissections or scanning electron microscopic investigations. Moreover, we carried out macula labeling in the 3D images with the aid of the borders of the furrows of the otolith housing the macula (in the case of the saccular and lagenar otoliths; Figure 3A-B), in part by using otolith dimensions (utricular otolith), and sites of innervation by the VIII^th^ cranial nerve. Although histological serial sections have the advantage that single cells can be identified, determination of macula delimitations is still difficult because of the smooth transition between macula and non-sensory epithelium (Figure 3C). In addition, as otoliths have to be dissolved during sample preparation for histology, the organic remains of the otolith can only in part be used as guideline for the identification of macula dimensions. In the case of the macula utriculi, delimitations of this sensory epithelium were easier to identify (Figure 8).

### Projection of 2D ciliary bundle orientation patterns on the 3D models of the maculae

**Figure 9:**
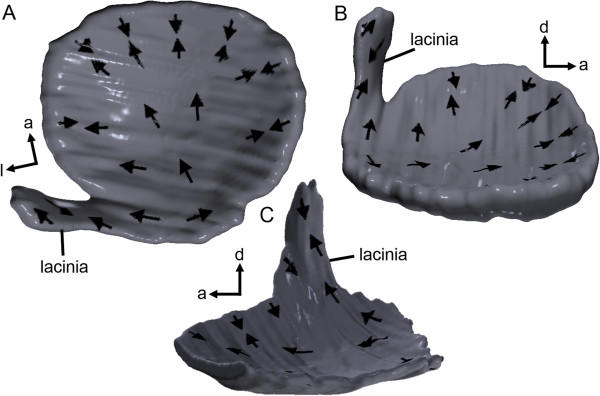
**Projection of the 2D orientation patterns of ciliary bundles onto the 3D models of the macula utriculi. (A-B)** Macula utriculi of *S. tinanti* in dorsal **(A)** and medial **(B)** views. **(C)** Macula utriculi of the black molly in medial view. Especially the strongly curved lacinia illustrates the value of orientation patterns shown in 3D. a, anterior; d, dorsal; l, lateral.

In both species the bowl-shaped macula utriculi and its laterally situated lacinia shows the advantage of the projection of the 2D orientation pattern of ciliary bundles on the 3D models. Orientation patterns can be studied from different perspectives following the 3D curvature of the macula (Figure 9).

3D models of maculae as obtained by our approach and subsequent projection of 2D evaluated orientation patterns of ciliary bundles of sensory hair cells onto the 3D reconstructed maculae (see Figure 9), may be especially important in comparative studies investigating several species of the same group with regard to their inner ear morphology and hearing abilities. Buran et al. (2005) [12] found distinctly out-of-plane curved maculae lagenae in one out of four studied elopomorph species. Ramcharitar et al. (2004) [13] reported strongly curved maculae sacculi in a sciaenid species (*Bairdiella chrysoura*); the authors [13] assumed that this may have an impact on the hearing abilities. Future application of microCT imaging on stained specimens in such studies will therefore have an impact in two ways: (i) peculiarities such as strongly curved maculae can be illustrated and compared qualitatively in 3D by the authors and the readers using interactive 3D models, and (ii) 3D datasets offer the possibility for a quantification of the amount of curvature of maculae, evaluation of the angle between the macula or otoliths to the sagittal plane, or to perform other 3D morphometrics (e.g., [14,15]), or even functional modeling of the sensory organs.

### Further future applications

So far, PTA contrasting for microCT has less frequently been used compared to iodine-based stainings for example. PTA staining provided good results for studying vertebrate embryos and thus is of great value for developmental studies [16,17].

Once the specimen is stained, microCT scans can be performed using different resolutions. Scans at “lower” resolutions (ca. 5-10 μm voxel sizes) may help to gain additional insights for example on the position and distance of inner ears to the swim bladder which is an important aspect in fish hearing; a small distance or even a connection between swim bladder and inner ears is likely to enhance auditory abilities (for an overview see [18]). High-resolution scans on the same specimen then allow for deciphering details of inner ear anatomy that yield not only information on the 3D curvature of maculae, but also on the in situ position and orientation of the maculae relative to each other and on the amount of overlap between macula and the corresponding otolith (see also Additional file 1, Additional file 2). The latter information may be of particular interest because parts of the macula not overlain by the otolith are hypothesized to be stimulated in a different way than the overlain regions, which is suggested to have physiological implications for hearing in fishes such as sound source localization [19].

Finally, as the method produces highly informative datasets in a rather short time, these can be used by scientists interested in the diversity of inner ear morphology in fishes asking taxonomical, phylogenetic, or ecomorphological questions. Evangelista et al. (2010) [20] have already performed a quantitative comparison of inner ears in sharks and rays, concluding that variation in ears could best be explained by taking into account phylogeny as well as feeding strategy; however, the study was based on 2D measurements. Measurements taken from 3D models of inner ears comparing different taxa may elucidate certain specializations in inner ear morphology and hearing abilities found in some species. In phylogenetic studies on inner ears in primates, such 3D morphometric analyses based on CT imaging have already successfully been applied (e.g., [21,22]).

Successful 3D reconstruction of fish inner ears has recently been performed using thin-sheet laser imaging microscopy (TSLIM) [23] (see also [24,25]). Though TSLIM provides excellent resolution similar to histological sections, a decalcification step during sample preparation is necessary. In the case of fish inner ears this means that otoliths have to be dissolved, i.e. information about otolith morphology and the relationship between otoliths and corresponding maculae is lost. A combination of both non-destructive methods [24], however, might further improve the success of 3D reconstruction of fish ears and their components.

## Conclusions

Our study shows that high-resolution contrast enhanced microCT imaging of PTA stained fish provides a powerful method to obtain reliable 3D images of fish inner ears including the maculae in a less labor-intensive way compared to most previous studies illustrating fish inner ears and/or maculae in 3D based on histological methods [2,4,26]. We suggest that future studies will benefit from using such 3D models of fish inner ears and maculae in comparative investigations focusing on functional morphology, hearing specializations and morphometric analyses. As dehydration and staining can differently affect inner ears of different species, any analysis of inner ears in 3D should include an investigation of the effects on the species under study.

## Competing interests

The authors declare that they have no competing interests.

## Authors’ contributions

TSM, MH, and BM conceived and designed the study. BM carried out the microCT imaging. TSM performed the 3D reconstructions and dissections. MH created the interactive 3D models. All three authors equally contributed to and wrote the manuscript. All authors read and approved the final manuscript.

## Supplementary Material

Additional file 1 **Voxel size in MicroCT imaging considerably affects accuracy of the 3D reconstruction of the lacinia (B: 2 μm isotropic *vs. *A: 5.4 μm isotropic).** The white arrow indicates the reconstructed lacinia of the macula utriculi. Brain (orange); c, cristae of the anterior (ca, green), horizontal (ch, yellow), and posterior (cp, blue) semicircular canals; lot, lagenar otolith (yellow); mu, macula utriculi (light brown); sot, saccular otolith (purple); uot, utricular otolith (red). Scale bars, 500 μm. The interactive 3D models can be accessed by clicking onto the figures (Adobe Reader Version 7 or higher re-quired). Rotate model: drag with left mouse button pressed; shift model: same action + ctrl; zoom: use mouse wheel (or change default action for left mouse button). For selection or deselection (or changed transparency) of components in the model tree, switch between prefab views or change surface visualization (e.g. lighting, render mode, crop etc.). Deactivate 3D content via context menu (right mouse click). Click here for file

Additional file 2 **Interactive 3D model of the left inner ear of *Steatocranus tinanti*.** c, cristae of the anterior (ca, green), horizontal (ch, yellow), and posterior (cp, blue) semicircular canals; lot, lagenar otolith (yellow); ml, macula lagenae (dark brown); ms, macula sacculi (yellow orange); mu, macula utriculi (light brown); sot, saccular otolith (purple); uot, utricular otolith (red). Scale bars, 1 mm. For activation of the inactive model see Additional file 1. Click here for file
