# Supplementary material for: Sensory epithelia of the fish inner ear in 3D: studied with high-resolution contrast enhanced microCT
Source: Front Zool. 2013 Oct 27;10:63. doi: 10.1186/1742-9994-10-63 (PMC4177137; doi:10.1186/1742-9994-10-63)
Supplement: Additional file 1 — Voxel size in MicroCT imaging considerably affects accuracy of the 3D reconstruction of the lacinia (B: 2 μm isotropic vs. A: 5.4 μm isotropic). The white arrow indicates the reconstructed lacinia of the macula utriculi. Brain (orange); c, cristae of the anterior (ca, green), horizontal (ch, yellow), and posterior (cp, blue) semicircular canals; lot, lagenar otolith (yellow); mu, macula utriculi (light brown); sot, saccular otolith (purple); uot, utricular otolith (red). Scale bars, 500 μm. The interactive 3D models can be accessed by clicking onto the figures (Adobe Reader Version 7 or higher re-quired). Rotate model: drag with left mouse button pressed; shift model: same action + ctrl; zoom: use mouse wheel (or change default action for left mouse button). For selection or deselection (or changed transparency) of components in the model tree, switch between prefab views or change surface visualization (e.g. lighting, render mode, crop etc.). Deactivate 3D content via context menu (right mouse click). [file 1742-9994-10-63-S1.pdf]

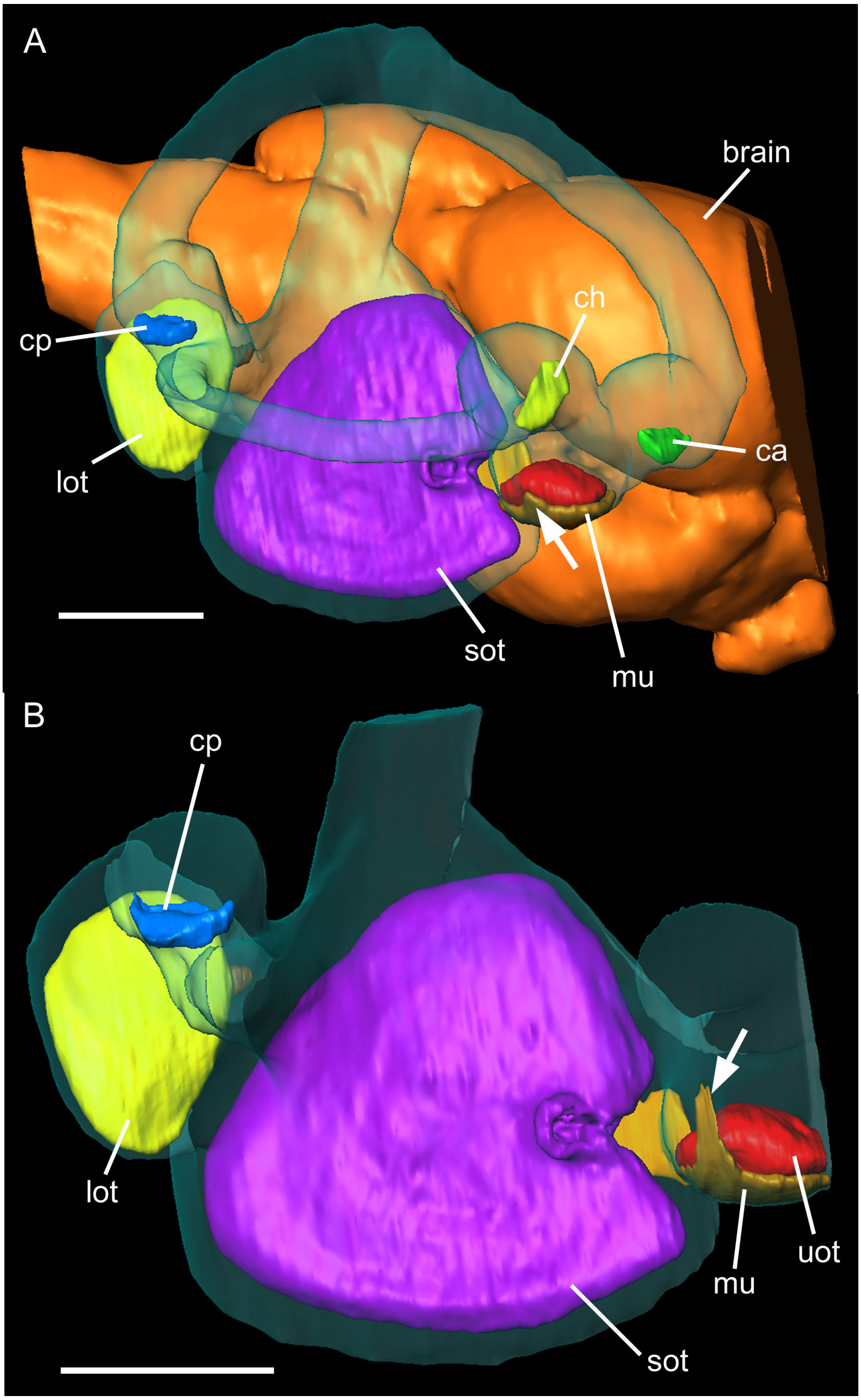

Additional file 1. Voxel size in MicroCT imaging considerably affects accuracy of the 3D reconstruction of the lacinia (B: 2 µm isotropic *vs.* A: 5.4 µm isotropic).

The white arrow indicates the reconstructed lacinia of the macula utriculi. Brain (orange); c, cristae of the anterior (ca, green), horizontal (ch, yellow), and posterior (cp, blue) semicircular canals; lot, lagenar otolith (yellow); mu, macula utriculi (light brown); sot, saccular otolith (purple); uot, utricular otolith (red). Scale bars, 500 µm. The **interactive 3D models** can be accessed by clicking onto the figures (Adobe Reader Version 7 or higher required). Rotate model: drag with left mouse button pressed; shift model: same action + ctrl; zoom: use mouse wheel (or change default action for left mouse button). For selection or deselection (or changed transparency) of components in the model tree, switch between prefab views or change surface visualization (e.g. lighting, render mode, crop etc.). Deactivate 3D content via context menu (right mouse click).
