# Supplementary material for: Sensory epithelia of the fish inner ear in 3D: studied with high-resolution contrast enhanced microCT
Source: Front Zool. 2013 Oct 27;10:63. doi: 10.1186/1742-9994-10-63 (PMC4177137; doi:10.1186/1742-9994-10-63)
Supplement: Additional file 2 — Interactive 3D model of the left inner ear of Steatocranus tinanti. c, cristae of the anterior (ca, green), horizontal (ch, yellow), and posterior (cp, blue) semicircular canals; lot, lagenar otolith (yellow); ml, macula lagenae (dark brown); ms, macula sacculi (yellow orange); mu, macula utriculi (light brown); sot, saccular otolith (purple); uot, utricular otolith (red). Scale bars, 1 mm. For activation of the inactive model see Additional file 1. [file 1742-9994-10-63-S2.pdf]

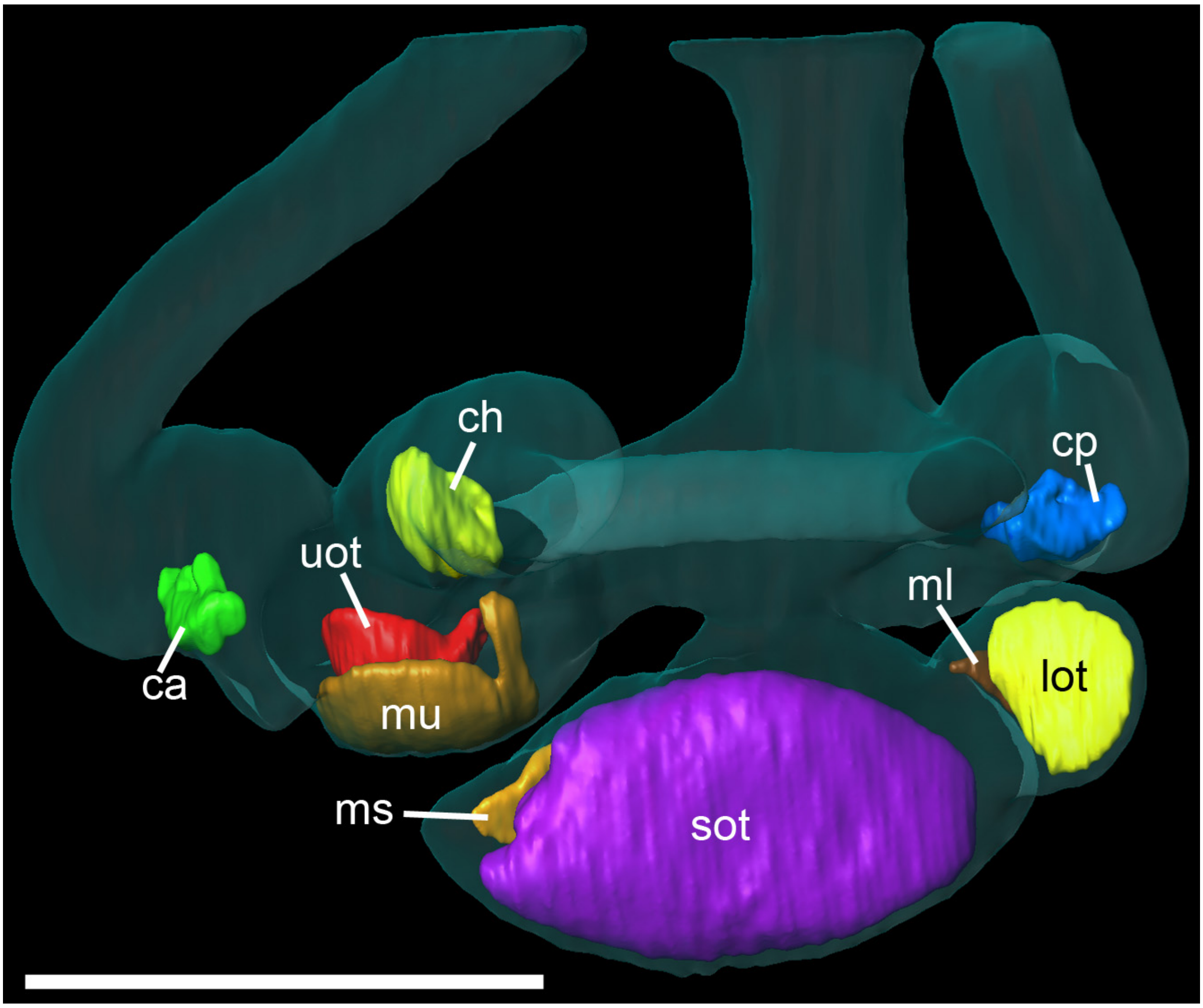

Additional file 2. Interactive 3D model of the left inner ear of *Steatocranus tinanti*.
c, cristae of the anterior (ca, green), horizontal (ch, yellow), and posterior (cp, blue) semicircular canals; lot, lagenar otolith (yellow); ml, macula lagenae (dark brown); ms, macula sacculi (yellow orange); mu, macula utriculi (light brown); sot, saccular otolith (purple); uot, utricular otolith (red). Scale bar, 1 mm. For activation of the interactive model see Additional file 1.
